# High Theta and Low Alpha Powers May Be Indicative of BCI-Illiteracy in Motor Imagery

**DOI:** 10.1371/journal.pone.0080886

**Published:** 2013-11-22

**Authors:** Minkyu Ahn, Hohyun Cho, Sangtae Ahn, Sung Chan Jun

**Affiliations:** 1 School of Information and Communications, Gwangju Institute of Science and Technology, Gwangju, South Korea; 2 Wadsworth Center, New York State Health Department, Albany, New York, United States of America; College of Mechatronics and Automation, National University of Defense Technology, China

## Abstract

In most brain computer interface (BCI) systems, some target users have significant difficulty in using BCI systems. Such target users are called ‘BCI-illiterate’. This phenomenon has been poorly investigated, and a clear understanding of the BCI-illiteracy mechanism or a solution to this problem has not been reported to date. In this study, we sought to demonstrate the neurophysiological differences between two groups (literate, illiterate) with a total of 52 subjects. We investigated recordings under non-task related state (NTS) which is collected during subject is relaxed with eyes open. We found that high theta and low alpha waves were noticeable in the BCI-illiterate relative to the BCI-literate people. Furthermore, these high theta and low alpha wave patterns were preserved across different mental states, such as NTS, resting before motor imagery (MI), and MI states, even though the spatial distribution of both BCI-illiterate and BCI-literate groups did not differ. From these findings, an effective strategy for pre-screening subjects for BCI illiteracy has been determined, and a performance factor that reflects potential user performance has been proposed using a simple combination of band powers. Our proposed performance factor gave an r = 0.59 (r^2^ = 0.34) in a correlation analysis with BCI performance and yielded as much as r = 0.70 (r^2^ = 0.50) when seven outliers were rejected during the evaluation of whole data (N = 61), including BCI competition datasets (N = 9). These findings may be directly applicable to online BCI systems.

## Introduction

Brain Computer Interface (BCI) technology provides a potential communication tool between the brain and a machine/computer, which would permit people carry out their daily lives with more convenience. However, there are certain hurdles to resolve before this technology can be offered on the public market. Depending on the control paradigm used, about 10-30% of BCI users reportedly do not modulate the brain signals that are required to run the BCI system. For example, 6.7% of subjects showed poor performance, with less than 59% accuracy in two-class motor imagery (MI) BCI [1]. In P300 BCI, 27.2% of subjects were unable to use the BCI system in a row/column paradigm and about 45% of subjects failed to achieve 100% accuracy in a single character speller test [2]. In a study that employed steady-state visual evoked potential (SSVEP) BCI with 37 subjects [3], approximately 14% failed to complete spelling tasks within the allotted time. Because this type of paradigm is widely used in BCI systems [4], BCI-illiteracy may be an obstacle to the practical application of this technology.

So far, there are several studies that compare BCI-illiteracy to BCI-literacy [5]–[10]. These studies reported that some neurophysiological differences exist between these two groups. Blankertz et al. [5] proposed an impressive sensory motor rhythm (SMR) predictor with a short resting state that can be applied prior to a time-consuming BCI experiment in order to evaluate a user’s potential performance. For MI-based BCI, the power decrease/increase at a specific frequency that is referred to as event-related (de)synchronization (ERD/ERS) has been employed for practical ends, which uses the fundamental characteristic of MI. This ERD/ERS phenomenon is explained in more detail by Pfurtscheller et al. [11]. The ERD usually occurs at alpha or beta bands, while ERS is visible at alpha or beta bands at a certain time or in a specific area of the cortex. Recently, the gamma band demonstrated more gain because Grosse-Wentrup et al. [12] reported the causal influence of the gamma band on SMR. Therefore, the gamma band is believed to affect both the subject and session variability, which are the primary areas of concern within the BCI community.

To the best of our knowledge, the theta band has not been adequately studied as part of the BCI-illiteracy phenomenon. The role of the theta band is known to be related to memory formation, information processing [13], working memory [14]–[16] and sensorimotor integration [17]. Moreover, the frontal lobe theta band was reported to reflect mental activity, personality traits and attention/arousal [18]. Therefore, there is good reason to further investigate the theta band in order to understand exactly how different the BCI-illiterate and BCI-literate users are, and whether or not any neurophysiological differences exist.

For this study, we investigated a mental state during a person is not involved in a certain task. This state is commonly used in the analysis of the default mode network (DMN) which is a kind of brain network [19]–[21]. Here, we introduced this state and named as NTS (non-task related state) throughout this study. Here, we introduced the NTS while subjects had their eyes open, as in Blankertz et al. [5]. We explain the experimental procedure, EEG acquisition and the methods that were used to evaluate BCI performance. Group divisions and mental state types are defined for further study. Then we investigate the difference between two groups in terms of NTS and spatial pattern changes over different mental states. We discuss how these investigations and their associated interpretations can be applied to quantify BCI-illiteracy potential, which may be of great use for pre-screening purposes before long BCI experiment is conducted. In addition, a concise BCI performance predictor is proposed here. The efficacy of these classifiers and predictors in determining BCI ability is verified by BCI competition 2008 datasets. Finally, other interpretations of the results are discussed.

## Materials and Methods

### Subjects

Fifty two healthy subjects (26 males, 26 females; mean age: 24.8 ± 3.86 years) were participated in this study. The experiment was approved by the Institutional Review Board of Gwangju Institute of Science and Technology. We informed all participants of the experimental purpose and process, and collected written consent letters.

### Experiment and EEG datasets

**Figure 1:**
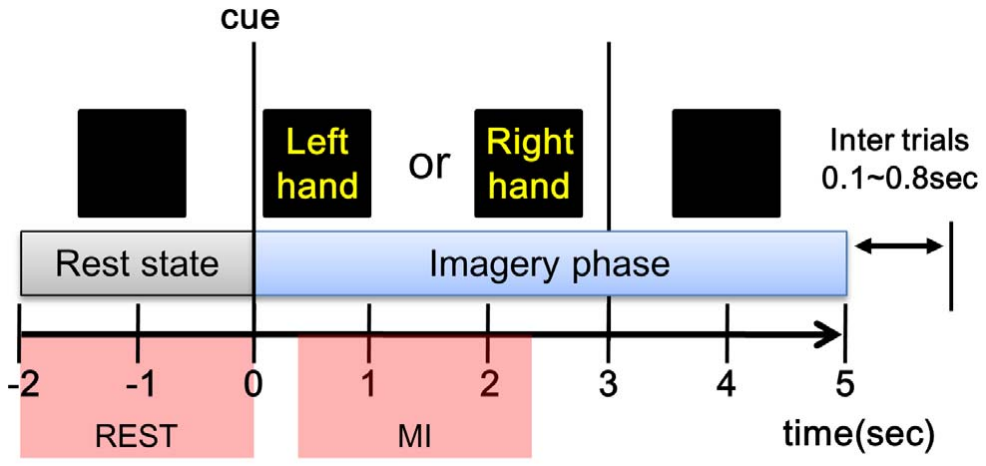
Procedure for one trial in an MI experiment.

Hand movement motor imagery experiment was conducted with every subject. For every experiment, BCI2000 software [22] and the Biosemi Active 2 system (64 active electrodes, 512 Hz sampling rate) were used to acquire EEG data. For our purpose, two kinds of datasets were acquired as follows:

- **Non-task related state.** EEG data were first acquired under the eyes-open condition for investigation of totally relaxed mental state. In the eyes-open condition, subjects do not conduct a task; instead, they let their minds wander or think about nothing. Therefore, throughout this paper this mental state is named as non-task related state and is abbreviated as NTS.
- **REST and MI states.** Next, five or six runs of a motor imagery experiment were conducted per subject. In this experiment, a conventional two-class motor imagery paradigm was applied; it required the imagination of left or right hand movements according to instructions that were presented on a monitor screen. For the first 2 seconds, subjects stared at the screen and imagined left/right hand movement when the instructions (cue) appeared on the screen for 3 seconds. This motor imagery step continued for a total of 5 seconds, including 2 seconds after the screen went blank. Figure 1 illustrates one trial of this motor imagery experiment. Subjects were given 20 trials for each condition (left or right motor imagery) per run. Thus, a total of 100 or 120 trials were collected for each MI condition. We divided each MI task into two phases: resting state (REST) before onset and MI state (marked in Figure 1). Since REST is closer to a task-involved state than NTS, we expected that NTS and REST might be different, so these two states were separated for comparative study.

### BCI offline performance and group categorization

Optimal or suboptimal frequency and temporal information for BCI data were varied by subject; that is, they were subject-specific. Some special methods may be applied to make these frequency determinations. In keeping with the simplicity of this work, we used an 8–30 Hz frequency band as an informative spectral interval. This spectral interval includes both alpha and beta bands, which have been reported to show motor imagery-related power increases or decreases [11]. We band-pass filtered each motor imagery dataset at 8 Hz to 30 Hz and then extracted time series data from 0.4 to 2.4 seconds after the onset, during which the ERD/ERS occurs at the time of motor imagery [11], [23]. The offline accuracy of the trials was estimated with the common spatial pattern (CSP) and Fisher linear discriminant analysis (FLDA) [23]. In general, cross-validation was introduced in order to yield a statistically reasonable BCI performance. In this work, a 10-fold cross-validation was applied as follows: every trial was grouped into ten sets; these ten sets were separated into 7 training and 3 testing sets. The number of possible separation is 120 cases. The BCI performance was estimated using 120 total types of separated testing and training sets. Finally, a mean accuracy of 120 estimates was assigned to each subject as his/her BCI performance.

With these estimated BCI performances, we attempted to identify BCI-illiterate subjects. Usually, these subjects perform at near-chance levels. Here, we set 60% of offline accuracy as a threshold because it is considered to be a roughly random performance with respect to the number of trials that were collected [24]. A minimum threshold for accuracy was needed for comparison. Some investigators might have chosen to use 60% again; however, we observed that the subjects who performed slightly better than 60% were yielding different information from those with very high performances. Therefore, we increased the minimum threshold for the high performance group to 70%. Finally, we assigned every subject to one of three groups:

- Group ‘A’ had 70% accuracy or above
- Group ‘B’ had 60% to 70% accuracy
- Group ‘C’ had lower than 60% accuracy

**Figure 2:**
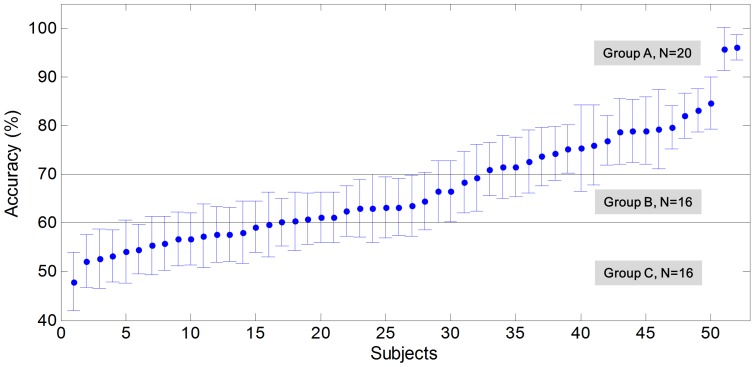
Estimated performance by CSP and FLDA. Subjects are assigned to groups ‘A’, ‘B’ or ‘C’ according to the accuracy of their performance.

This study especially focused on Groups ‘A’ and ‘C’ to pinpoint the differences between subjects who were rated as BCI-illiterate and those rated as BCI-literate. Group ‘B’ was rated as in-between. In Figure 2, the performances of the subjects in each group are noted with their standard deviations.

### Preprocessing for spectral analysis

This data allowed us to compare two categorized subject groups: group A (BCI-literate) and group C (BCI-illiterate). We wanted to find if there were any substantial neurophysiological differences between the two groups. For this purpose, we considered three different mental states: NTS, REST and MI states. For each mental state, the notable neurophysiological characteristics of the two subject groups were thoroughly investigated.

For the first step, we preprocessed EEG datasets as follows: to remove noise coming from the electric power, the EEG datasets were notch-filtered at 60 Hz and re-referenced using a common average reference, but no noise rejection procedure was applied. Depending on the source of information, the designated frequency bands of interest are, in general, moderately varied. Therefore, we decided to use frequency ranges from theta (4–8 Hz), alpha (8–13 Hz), beta (13–30 Hz) and gamma (30–70 Hz) frequencies for spectral power analysis. Here, the delta band at around 1–4 Hz has been excluded because it is contaminated relatively easily by artifacts such as eyeball movement, blinking, jaw movements and a certain degree of electromyogram noise. On the other bands, the spectral powers were estimated by using a fast Fourier transform on the time series for each channel with an EEGLAB library [25]. However, the powers in each band were very different across the subjects, thereby making it difficult to see a pattern at the group level. To minimize this problem, we normalized the band powers by using the total power, which was obtained by summing all powers from 4–70 Hz. Comparative analysis across subjects was facilitated by using this method, and throughout this study, this resulting value is called the relative power level (RPL).

The REST and MI states were preprocessed in the same manner, and RPLs were computed for analysis. These RPLs were used to find statistical differences between the BCI-literate (group A) and BCI-illiterate (group C) groups.

## Results

### Characteristics of NTS between BCI-literate and BCI-illiterate groups

**Figure 3:**
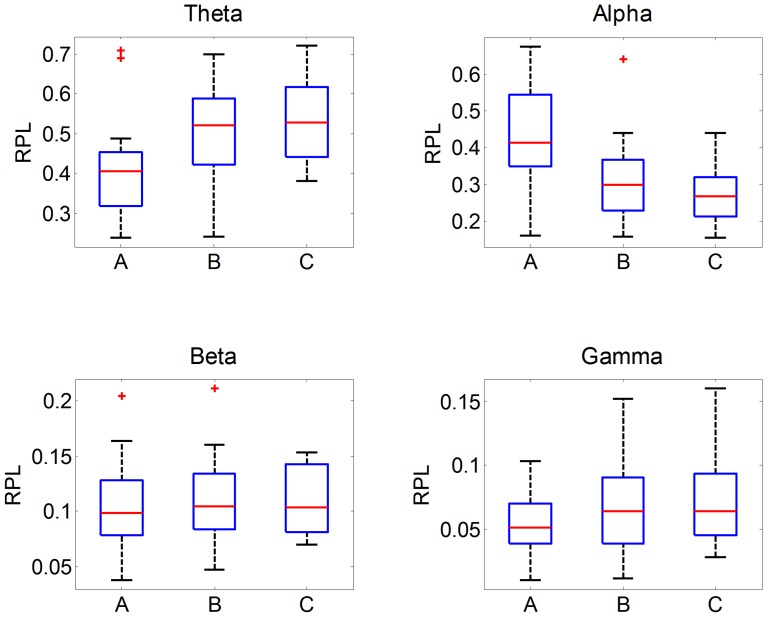
The mean distribution of RPL over all channels for each band and each group. This result is obtained from the NTS signal and whisker length [52], which is set to 1.5. The outliers (red crosses) are categorized on the basis of the whisker lengths. For results from statistical test, please see the section ‘Characteristics of NTS between BCI-literate and BCI-illiterate groups’.

To examine the statistical differences between the BCI-literate and BCI-illiterate groups, the distributions of mean RPL values over all channels for each categorized group were presented in a box plot; some outliers based on whisker length of 1.5 were denoted by a red ‘+’ as shown in Figure 3. Wilcoxon rank-sum test [26], [27] was conducted to investigate whether or not a pair of distributions are statistically different, that is, whether or not null hypothesis (two distributions are statistically the same) is rejected. As a result, we observed that two group pairs AB and AC are significantly different (significance level p < 0.05) in both theta and alpha band powers. However, group pair BC in theta/alpha band powers and all group pairs in beta/gamma band powers did not show statistical significance.

**Figure 4:**
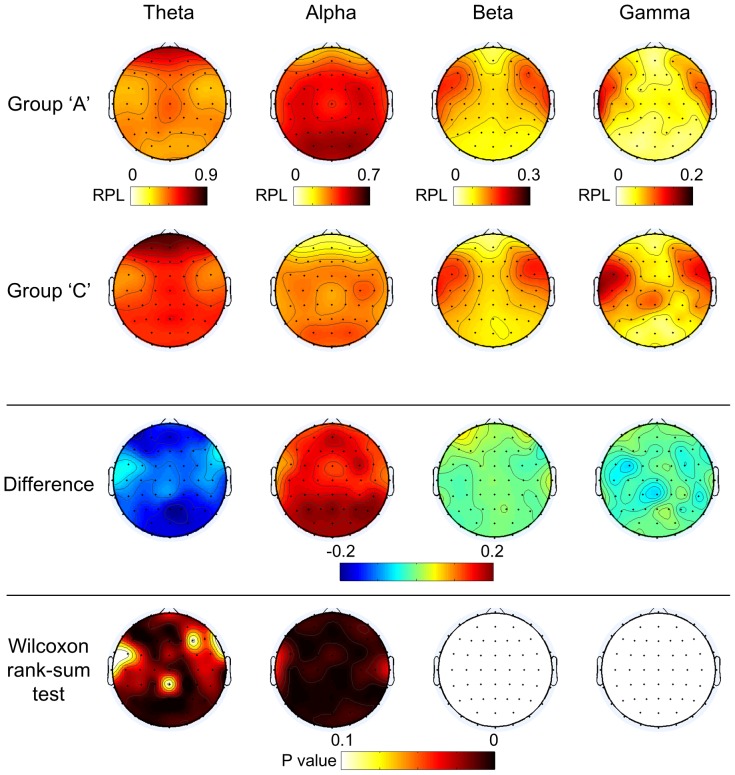
Spatial distributions of RPL for NTS over various frequency bands. The comparison of NTS (1^st^ and 2^nd^ rows), their differences (the 3^rd^ row was calculated by subtracting group C from group A), and the result of a Wilcoxon rank-sum test (FDR corrected) between the 1^st^ and 2^nd^ rows (4^th^ row)

The overall difference between subject groups was not that significant for beta and gamma. However, the tendency for theta waves to increase and alpha waves to decrease was noticeable in the RPL distribution, which could be observed when comparing group A and group C. The spatial distributions of RPL magnitudes for NTS over the subjects and frequency bands are presented for groups A and C in Figure 4. The overall distribution of the four frequency bands was similar for all subject groups. The theta level was relatively high in the prefrontal and central areas near Cz. The alpha levels were high in the occipital lobe and decreased from the occipital to the prefrontal area. High levels of beta and gamma were observed near frontal-temporal channels. However, some differences can be observed easily when comparing the different groups. Interestingly, the theta power level was lower and the alpha power level was higher in group A than those in group C. Beta levels were slightly higher for group A than group C, except in the occipital lobe, while group C showed slightly higher gamma levels. The third row in Figure 4 marks the difference between the first and second rows, and the fourth row is spatial distribution of p-value. Here Wilcoxon rank-sum test was applied (False Discovery Rate (FDR) corrected with q = 0.05) [28], [29].

From the different topographical images in the third row in Figure 4, it becomes clear that group C has a higher theta power and lower alpha power than group A, with statistically significant differences between the frontal and posterior-parietal cortex for theta waves, roughly across entire area for alpha waves. However, the beta and gamma powers did not have a spatially significant difference. These observations are briefly summarized in Table 1.

**Table 1:** Summary of comparison between groups A and B.

	Theta	Alpha	Beta	Gamma
Group A	Low	High	A little higher in frontal and central	
Group C	High	Low		A little higher in the temporal and posterior-parietal areas
Wilcoxon rank-sum test	Frontal, Posterior-parietal	Roughly across entire area		

### Characteristics of REST and MI

**Figure 5:**
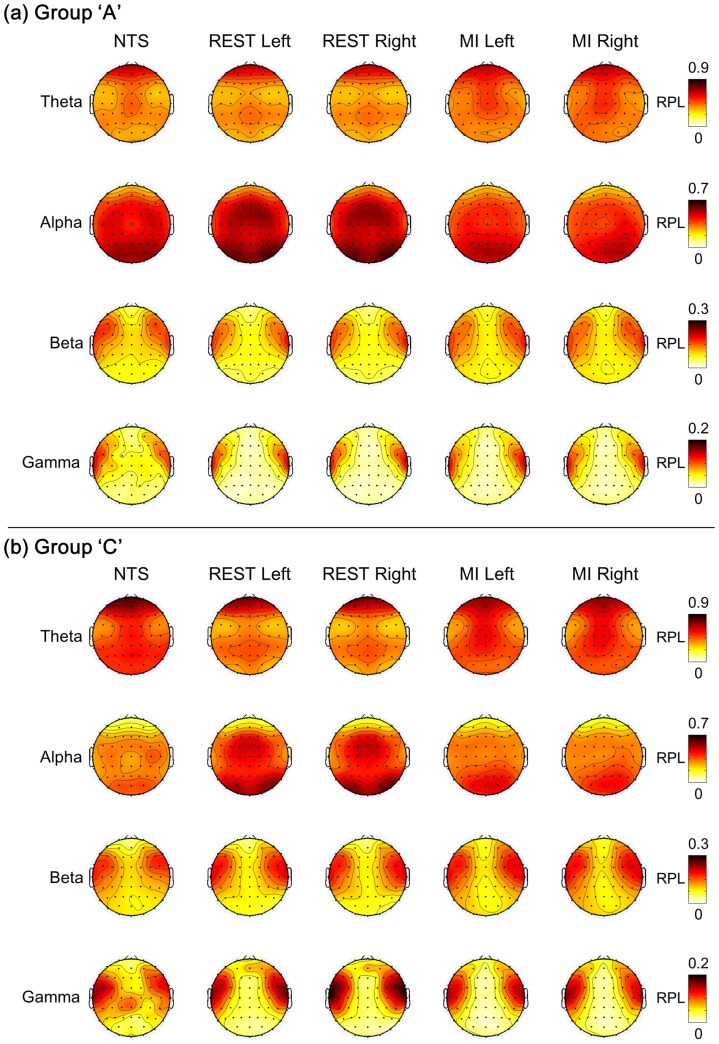
Spatial patterns (group averaged) of RPL over three different mental states (NTS, REST and MI).

We have compared the following three different states: the NTS, REST and the MI state. Figure 5 shows the results of RPL for three different states. In the previous section, we observed relatively higher theta and lower alpha bands in group C than in group A. Here, this behavior was still sustained, but the pattern changed according to the mental states. First, the RPL for the theta band decreased from NTS to REST, while the RPL for the alpha band increased in the mid-frontal and lateral-occipital areas. However, this phenomenon was not noticeably observed in MI. Conversely, the RPL for the theta band in MI showed a new pattern (different from REST) in which high RPL appeared from the pre-frontal area to the mid-central area. Looking at the beta and gamma bands, overall patterns were similar for all three mental states, but we observed a slight RPL magnitude increase when NTS moved to REST and MI. Our findings may be summarized as follows:

- In group C, the pattern of relatively high theta and low alpha bands was sustained over all three different states (NTS, REST, and MI).
- In both groups A and C,
  - For both REST and MI states, there was no substantial difference between the left and right hand movements.
  - The theta power decreased when moving from NTS to REST, while the alpha power increased with a focus on the mid-central and lateral-occipital areas.
  - When moving from REST to MI, the high theta RPL seemingly moved from the pre-frontal area to the mid-central area, but the high alpha RPL disappeared.
  - The RPLs for beta and gamma bands slightly increased from NTS to REST and MI.

From these findings, we believe that the three states (NTS, REST, MI) are mentally different, even though both groups A and C showed similar patterns when the three states were propagated (from NTS to REST to MI). We noted that these findings were similar to those observed in the ERD/ERS analysis (not shown here). Further interpretation is discussed later.

### Relationship between BCI performance and spectral band power

**Figure 6:**
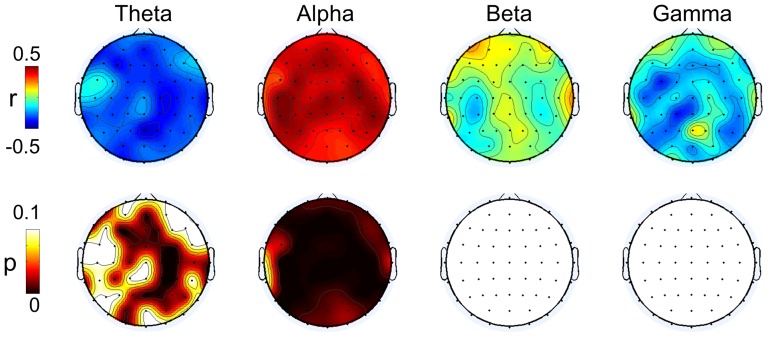
The result of correlation analysis between spectral band power and performance. Upper row represents correlation coefficient and lower image shows corresponding p-value (FDR corrected) for four bands.

We have observed differences in the RPL distribution over three mental states between the BCI-literate and BCI-illiterate groups. In this section, we investigated how much each spectral band is related to the BCI classification performance. These results may motivate us to examine the spatial and spectral correlation distributions with BCI performance more thoroughly for motor imagery data. The Pearson correlation coefficients [26] which measure linear correlation between RPL (over channels and spectral bands) and BCI performance were computed. Figure 6 shows the spatial distributions of the Pearson correlations and corresponding p-values (FDR corrected with q = 0.05) between the RPL for each band and the BCI performance. The theta band power had an overall negative correlation with BCI classification accuracy in the region, while the alpha band power was positively correlated and seemed far more strongly correlated around C3 and C4. High alpha power near sensory-motor rhythm was reported in Blankertz et al. [5], which was used as a performance predictor in MI BCI. In addition, the beta and gamma powers were relatively less correlated with BCI performance and its significant level is very lower showing the high p-value. Although the statistical test does not result significance, the gamma power had a weakly negative correlation in the centro-parietal area; this data is relevant to the finding about the causality of gamma on SMR [12]. These authors also demonstrated that the gamma band reflects the attention process, and so it may be shifted from the centro-parietal to the frontal and occipital areas. Therefore, this process can influence the MI step.

From these observations, we can infer that the theta and alpha bands may offer more information than the beta and gamma bands to explain the user’s MI ability. From Figure 6, the correlations for theta and alpha increased up to |r| = 0.50, while those from the beta and gamma bands were less than |r| = 0.40 (maximum correlations: |r| = 0.26 for beta and |r| = 0.37 for gamma). The statistical significance levels by p-value in theta and alpha are high enough to conclude. In short, theta and gamma were negatively correlated, while alpha and beta were positively correlated with BCI performance.

### Applicability of high theta and low alpha patterns

So far, we have observed that relatively higher theta and lower alpha power patterns are typical in BCI-illiterate subjects in comparison to BCI-literate subjects. This finding may be broadly applicable. One intuitive application is to generate a strategy to discriminate between specific users with high BCI-illiterate potential. In a similar manner, it is also possible to model various performance predictors that incorporate high theta and low alpha patterns. These applications should be highly beneficial in that they provide a solid pre-screening methodology (depending on the investigator’s use) and even a rough performance prediction for each user before time-consuming experiment is conducted. In general, there is substantial subject-to-subject and even trial-to-trial mental state variations, which makes it difficult to develop a robust BCI system. With these patterns it is now possible to determine whether or not a current mental state is good enough for developing a BCI control. According to such mental state decoding, users can do a reasonably good job of using a BCI system. In this section, we propose an effective pre-screening strategy and a performance predictor from our existing datasets. BCI Competition 2008 datasets 2b, which are commonly used in the BCI community, were used for verification purposes.

**BCI Competition 2008 datasets.** For our purposes, we introduced additional datasets from BCI competition 2008. These datasets are EEG data from 9 subjects in MI experiments (left/right hand movement imagination). Every subject conducted three training sessions and two feedback sessions. Only three channels (C3, Cz and C4) were available with a sampling rate of 250 Hz. The signals were analog band pass filtered (0.5–100 Hz) and notch filtered at 50 Hz. The goal of this competition was to classify a user’s intention (for example, left or right hand) during two feedback sessions. For more details, please refer to [30]. It was reported that the first place winner’s mean kappa coefficient was 0.57. This kappa coefficient (κ) was obtained as follows:
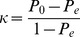
(1)


The P_0_ is the classification accuracy for BCI performance and P_e_ denotes the hypothetical probability of chance agreement. We used this winner’s kappa coefficient as an online performance measure for each subject. We noted that 7 out of 9 subjects showed reasonable good performances and the remaining 2 subjects were regarded as low performance or BCI-illiterate persons. Although Leeb et al. [30] reported offline BCI performances, the kappa values of the BCI competition winner showed a high correlation (r = 0.92) with the online motor imagery BCI performance test that was conducted in an immersive virtual environment (iVE). Notably, the competition winner’s performance was slightly higher than that of the online iVE BCI; thus we simply used it as an achievable maximum BCI performance. In this work, the RPL analysis was applied to the resting state signal under the eyes-open condition from the 5th session (last feedback session).

**Screening of BCI-illiterate subjects.** In this section, we attempted to apply our findings to discriminate between subjects with high BCI-illiterate potential from any given subject pool, which is of great use in the BCI research community. Prior to conducting a time-consuming BCI experiment, a brief acquired resting state can be used as a pre-screening strategy to infer how probable it is that a subject is BCI-illiterate. For this purpose, the RPLs of theta and alpha frequencies were extracted from each subject as a two-dimensional feature point and used to generate a classifier from these feature points that separate the high performance group (group A) from the low performance group (group C). For pre-processing, the outliers (subjects s6, s37 in group A) who showed unreasonably high theta RPLs were discarded according to statistical behavior (whisker length), as illustrated in Figure 3; these two subjects were marked with a red cross in Figure 3.

**Figure 7:**
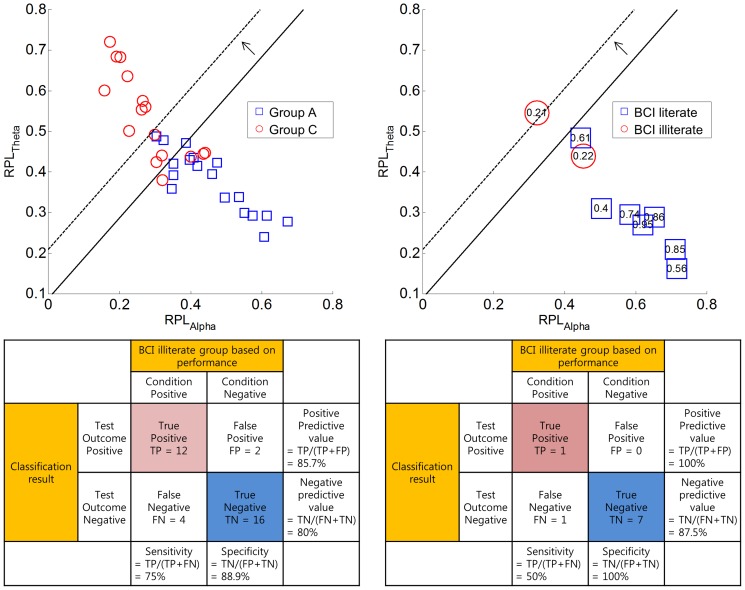
A group classification between BCI-literate (blue square) and BCI-illiterate (red circle) groups (Top left). It is shown with the discriminant line that was obtained by FLDA. The group classification line was applied to the BCI competition dataset in the top right figure. These numbers indicate the kappa coefficients for each subject and the confusion matrices are noted on the bottom line.

The FLDA was again applied here. The estimated discriminant line and the distribution of feature point are depicted on the left in Figure 7. The classification accuracy was 82.35% (or 72.22% when two outliers were not excluded). For the verification of our classifier, the BCI competition datasets that were explained in the previous section were used to extract features in the same manner as in our datasets. Figure 7 (top right) shows the distribution of feature points for nine subjects. Each feature point was labeled as a kappa coefficient, which is an indicator of BCI performance, implying that a low/high kappa coefficient represents low/high BCI performance. For example, when κ = 0.21 or κ = 0.22 in Figure 7 (top right), this low value represents a low performance. In estimating the kappa coefficients by formula (1), we used P_e_ = 0.50 (as a statistical chance level in a two-class problem) since the exact P_e_ from a confusion matrix is absent.

The performance group (BCI-illiterate or BCI-literate) classifier that was generated from our offline dataset seemed to classify BCI competition data reasonably well, as shown in Figure 7 (top right). Among nine subjects, eight were classified as BCI-literate, while one was placed in the BCI-illiterate group. According to the results from the winner of the BCI Competition, seven out of nine subjects showed moderately good BCI performances. Two subjects with low kappa were likely to be BCI-illiterate. We tabulated the confusion matrix for this classification in Figure 7. In classifying BCI-illiterate subjects, it is better to be more conservative in order to reduce false positive cases (falsely categorizing a BCI-literate subject as BCI-illiterate). Even our classification results for the BCI competition data gave zero false positives. At the user’s discretion, more conservative classifiers were used to reduce false positives, moving the original discriminant line up slightly in a perpendicular direction to the original line; this trend is depicted as a dotted line in Figure 7. We observed that the classifier that was generated by 52 subjects (without exclusion of two outliers) resulted in the same difference.

**Prediction of potential performance.** In this section, we propose a simple BCI performance predictor as an additional application of our findings; typically, BCI-illiterate subjects tend to have relatively high theta, low alpha, low beta, and high gamma frequencies. As a result, by simply taking the relationships into account in one formula as follows, we may use proportional or inverse proportional relationships for RPLs with BCI performance, as we concluded in the section ‘Relationship between BCI performance and spectral band power’:
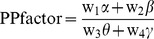
(2)


**Figure 8:**
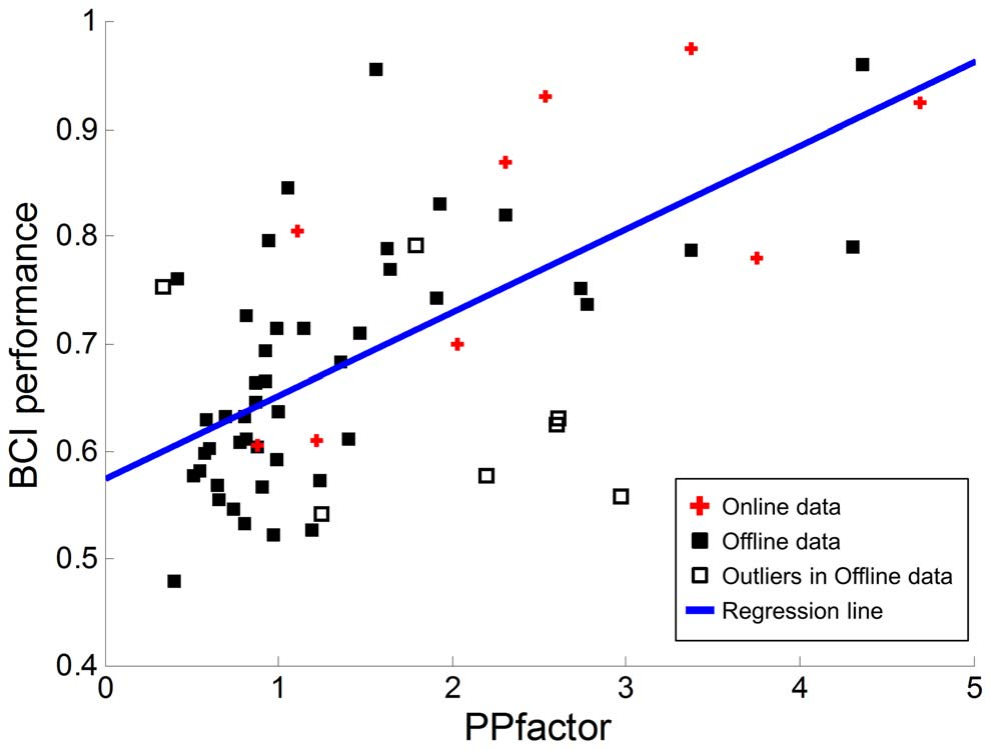
The relationship between the proposed PPfactor and BCI accuracy. The whole data points (N = 61) give a correlation value (PPfactor and BCI accuracy) of r = 0.59 (r^2^ = 0.35, P<1.0e-6). The association reaches up to r = 0.7 (r^2^ = 0.49, P<5.0e-9) when the rejection of seven outliers are applied. The regression line calculated from offline data excluding seven outliers is overlaid.

We refer to this relationship as a performance potential factor (PPfactor). It includes the RPLs for each of four spectral bands (alpha, beta, gamma, and theta) and corresponding control parameters (w_i_), which may be heuristically determined. To test this PPfactor, the RPLs from the C3 and C4 channels were used for every dataset (our offline and online data from the BCI competition). We observed the distributions of RPLs over average powers on C3 and C4 channels (for alpha, beta, gamma and theta bands) for 52 subjects. Seven subjects (s12, s22, s33, s35, s37, s39 and s45) showing unreasonably high RPLs (determined by whisker length of 1.5) were considered as outliers. The following analysis would be done for two cases (with outliers and without outliers). We set all the control parameters to w_i_ = 1 for the sake of simplicity. The relationship between the PPfactor and BCI performance are presented in Figure 8. The filled and blank squares denote our 52 subjects (offline dataset); these yielded a correlation of r = 0.48 (r^2^ = 0.23, p<5.0e-4) with BCI classification accuracy. The red crosses represent the online dataset from BCI competition data, which yielded a higher correlation of r = 0.69 (r^2^ = 0.48, p<0.05). For online BCI competition data, the classification accuracy was calculated by (1) and addressed in the previous section (assuming P_e_ = 0.50). The correlation value showed that r = 0.59 (r^2^ = 0.34, p<1.0e-6) for all datasets (N = 61, which consists of 52 our offline datasets and 9 BCI competition’s online datasets). If the rejection of seven outliers (unfilled circles in Figure 8) was applied, the correlation values were increased up to r = 0.64 (r^2^ = 0.41, p<5.0e-6) for offline datasets (N = 45) and r = 0.70 (r^2^ = 0.50, p < 5.0e-9) for the whole datasets (N = 54).

In addition, a regression model was generated to evaluate the predictability of BCI classification accuracy for the given PPfactor. For this purposed, a linear regression model was determined as a reasonable one after various regression models were tested. This regression model was cross-validated by leave-one-subject-out (LOO) method for 52 offline subjects. For each iteration, one subject was used as a test data and the remaining (51 subjects) was used to generate a linear regression model. The difference (root mean square error (RMSE)) between predicted performance by the regression model and actual BCI performance was calculated. A total of 52 iterations were done and it resulted in RMSE of 0.086 (RMSE  =  0.096 with outliers) for train data and 0.090 (RMSE  =  0.100 with outliers) for test data, respectively. Then, this linear regression model was applied to online data (BCI competition data). The prediction error (RMSE) was 0.101 (RMSE  =  0.120, tested by regression line generated from our data with outliers), which is slightly higher than RMSE for offline data. Figure 8 depicted the linear regression model estimated by all 45 offline subjects (without outliers) along with all offline and online data. This shows that our concise linear regression model reasonably well fits even online data and may be applicable to the prediction of BCI performance.

From these results, it is clear that even this simple PPfactor (as estimated from C3 and C4 only) could moderately predict a user’s potential performance. This method uses four kinds of band powers, which makes it easy to implement and apply. The combination of the PPfactor with other existing factors or more channels will facilitate better BCI performance prediction.

## Discussion

### Theta and alpha waves for motor imagery

During motor imagery, the power decrease (ERD) in alpha and beta bands is well known and is considered a promising feature of most MI-based BCI systems. The role of ERD in the alpha band is interpreted as a reflection of target motor cortex activation [11]. Therefore, potential decreases from the resting state could be used to understand a user’s performance on an MI-based BCI system [5]. Similarly, there have been some reports of theta band increases in motor imagery [14], [17] and working memory tasks [15]. Therefore, it is plausible that potential increases in the theta band reflect a user’s performance just as in the alpha band power. Our results were consistent with this reasoning and showed a positive correlation between offline accuracy in the alpha band power and a negative correlation in the theta band power. It was reported that the theta wave plays a role in such tasks as motor imagery, sensory-motor integration and memory load in rodents as well as humans [31]. Thus, the theta and alpha waves can be considered to reflect a user’s suitability for a MI-based BCI system.

### The influence of attention

We observed that the spatial pattern changed over the three mental states under study, that is, NTS, REST and MI. The theta band decreased in RPL during REST and MI, while the power level of beta and gamma bands increased. This simultaneous increase and decrease phenomenon may be relevant to the attention process. Whatever the mental state, either REST or MI, a certain degree of attention is required; therefore, a subject tries to prepare for the incoming instruction message in REST and imagine movement in MI. By doing so, the increases and decreases described above occur, reflecting the subject’s attention process. This hypothesis is strongly supported by several articles. In studies on attention deficit hyperactivity disorder (ADHD) [32], [33], theta suppression and beta enhancement were introduced for biofeedback. High frequency oscillations such as gamma have been associated with the attention process [34]–[37]. Thus, we expect that theta power may have a negative correlation with attention, while high oscillation bands may have positive correlations with a user’s attention level, as presented in our results.

### Alpha power increases during preparation

As shown in the section ‘Characteristics of RET and MI’, a visible change occurred in the alpha band during REST; it was focused in the mid-central and lateral-occipital areas. This mid-central area is near the pre-motor cortex, which area is related to motor planning [38], [39]. During REST, a subject was preparing for incoming instructions and was about to imagine his or her hand movement. Thus, the alpha power increase may be associated with the subject’s preparation for motor initiation.

In addition, another visible area of alpha power increase is in the occipital lobe; this area is separated into the left/right hemispheres. The alpha power increase in the occipital area is clearly related to the action of subjects, who were staring at the monitor and waiting for the instructions to appear on the screen. The alpha power might be increased by visual processing; however, it is separated into two parts. This may be understood as the attention process for the left and right hemispheres. There are some studies in which covert visuo-spatial attention shows spatially different alpha oscillations in the occipital lobe [40]–[42]. To perceive the direction in which to move the hand, the subject must integrate the direction from visual stimuli and execute the imagination of hand movement and the ready state may affect this alpha power increase in the lateral-occipital area. However, this phenomenon should be investigated further.

### BCI performance Prediction Factors

A user’s BCI performance is affected by various factors, such as the user’s ability to operate the BCI system successfully, his or her mental state, the classifier, feature extractor, and hardware issues. In this study, we focused on a user’s ability to use BCI; therefore, it may not predict a user’s potential performance perfectly, even when an ideal neurophysiological factor is constructed. An SMR predictor was proposed by Blankertz et al. [5] that reported an r = 0.53 (r^2^ = 0.28) correlation with BCI performance, while our method yielded slightly higher correlation values of r = 0.59 (r^2^ = 0.34) for all datasets (N = 61). The SMR predictor was designed with mainly µ (10–14 Hz) band information; however, our proposed PPfactor was designed with more frequency bands, including the μ. With our proposed method, it is still possible to show improvement by tuning the weighting of the channels or band powers, or even by introducing estimated powers in shorter frequency intervals. These weights and frequency intervals may be selected in an optimized manner and with more datasets that are acquired from many subjects. Our proposed potential factor in the section ‘Prediction of potential performance’ includes four simple kinds of band power factors that show negative or positive correlations, which may influence subject variability or even session variability performance. It is expected that this simple approach may be used to reduce the variability across both sessions and subjects, which is currently under investigation.

In addition to our findings so far (RPLs of alpha and theta), we found that calmness, ease of motor imagery and the subject’s expected performance were substantially statistically significant for BCI performance (not shown here) from questionnaires on the subject’s mental states before/after the experiment. This finding indicates that subject self-assessment may, to some extent, be helpful in predicting the subject’s performance.

### Cause and solution

Some may question why BCI-illiteracy occurs and what influences this phenomenon. These issues must be understood, as low performance is a fatal problem for BCI as it currently exists. With respect to the cause of BCI-illiteracy, Blankertz et al. [43] reported that the EEG is very sensitive to noise and is unable to detect sources in cortical folds. Therefore, a sensor cannot read the informative modulation of interesting sources, thereby resulting in a poor signal-to-noise ratio in some paradigms. Also, it was reported that the BCI-illiterate group demonstrates a higher noise than the BCI-literate group [8]. From this anatomical viewpoint, individual inter-hemispheric connectivity traits were also reported as one of the influences on the MI ability in a study of corpus callosum white matter [44]. On the other hand, there are also studies of psychological factors; a study on locus of control (LOC) showed that the LOC resulted in a correlation coefficient of r = 0.59 with respect to the technology and hit-rate [7]. The performance level, which was considered to be evidence of the degree of concentration, was identified as being significant [9]. Apart from these anatomical and psychological factors, another possible reason for BCI-illiteracy is that a user who does not show the suitable modulation may have an inadequately-trained neuronal network. For the MI paradigm, the questionnaire used to assess the subject’s ability in kinesthetic and visual imagery showed that the ability to produce imagery was relevant to estimating BCI performance [6]. Lastly, Halder et al. [10] insisted that the number of activated voxels in the brain might be fewer in the illiterate than in the literate group.

Another compelling issue is how to overcome the problem of BCI-illiteracy. Other existing paradigms than MI-based BCI can be used if the main cause for BCI-illiteracy is the anatomical structure or something that makes it difficult for a user to generate the detectable modulation. Thus, using different BCI control paradigms may be a possible solution as a BCI wizard to find a user-specific paradigm that can be used for better performance [45]. On the other hand, hybrid approaches may be introduced, since these methods facilitate the use of information from more than two paradigms [46]–[48]. In addition, other adaptive algorithms [49] or training-based approaches could be used to overcome low performance if the main reason is psychological factors or inadequately-trained brain networks [14], [50]. Biofeedback before using a BCI system is another solution, as biofeedback may shift the user’s mental state to one that is suitable for the BCI system. For example, gamma oscillation, which has a causal influence on SMR, was used to enhance the resting-state SMR by intentional attenuation of fronto-parietal gamma power during biofeedback [51].

In this section, we have reviewed reports about the possible reasons for the BCI-illiteracy phenomenon in addition to proposed solutions. However, a crucial cause, a promising solution, or even a treatment has not yet been proposed. Until BCI-illiteracy is fully understood, a special diagnosis for a user’s ability to run a control paradigm or pre-screening for BCI-illiterate users would obviously be beneficial to the BCI community. This information would probably help us to understand a user’s state and find a user-specific solution. There may be various factors that are helpful in predicting a user’s state, for example, when a user can perform well (and how well) or whether or not system environment/settings are reasonably good, etc. In this sense, the classification and simple PPfactor that is proposed in the section ‘Applicability of high theta and low alpha patterns’ could be used effectively along with other factors such as the psychological [7], [9], neurophysiological [5], system-relevant [45] or other self-assessed user factors [6].

## Conclusions

In existing BCI systems, 15–30% of target users (called ‘BCI-illiterates’) are known to show far poorer performances than others. BCI-illiteracy is an issue that needs to be understood in order for BCI systems to be useful in the future. In this study, we investigated the difference between BCI-literate and BCI-illiterate groups in terms of spectral band powers by comparing NTS during the eyes-open state, resting but ready state before motor imagery and motor imagery. With the motor imagery EEG datasets from 52 subjects, we found that the BCI-illiterate group showed high theta and low alpha power levels in comparison to the BCI-literate group. Statistically significant areas were distinguished as frontal and posterior-parietal regions for the theta band and the whole brain area for the alpha band. This high theta and low alpha pattern was sustained during other mental states such as resting before onset and motor imagery. However, this spatial pattern for each frequency band changed over varying mental states. These changes are considered to represent attention, motor-related memory load processes, and preparation for incoming instructions for the motor imagery phase. These spatial pattern changes were observed to be similar in both groups. By using the theta and alpha RPL from user resting state data, an effective strategy to discriminate between users with high BCI-illiterate potential (or high BCI-literate potential) was proposed. In addition, a simple performance predictor was proposed that used these neurophysiological findings and gave higher Pearson correlation coefficient values than the SMR predictor [5].

In conclusion, a pattern of high theta power and low alpha power may reflect BCI-illiteracy during the NTS. This finding could be used as the physiological factor, together with other possible factors, to understand a user’s potential ability to use a BCI system.
